# Toward Self-Powered Sensing and Thermal Energy Harvesting in High-Performance Composites v*ia* Self-Folded Carbon Nanotube Honeycomb Structures

**DOI:** 10.1021/acsami.3c08360

**Published:** 2023-09-11

**Authors:** Kening Wan, Arnaud Kernin, Leonardo Ventura, Chongyang Zeng, Yushen Wang, Yi Liu, Juan J. Vilatela, Weibang Lu, Emiliano Bilotti, Han Zhang

**Affiliations:** † School of Engineering and Materials Science, Queen Mary University of London, Mile End Road, London E1 4NS, U.K.; ‡ Department of Materials, Loughborough University, Loughborough LE11 3TU, U.K.; § IMDEA Materials Institute, Eric Kandel 2, Getafe 28906, Madrid, Spain; ∥ Division of Advanced Nanomaterials and Innovation Center for Advanced Nanocomposites, Suzhou Institute of Nano-Tech and Nano-Bionics, Chinese Academy of Sciences, Suzhou 215123, PR China; ⊥ Department of Aeronautics, Imperial College London, Exhibition Road, London SW7 2AZ, U.K.

**Keywords:** thermoelectric, carbon nanotubes, temperature-induced self-folding, self-powered sensing

## Abstract

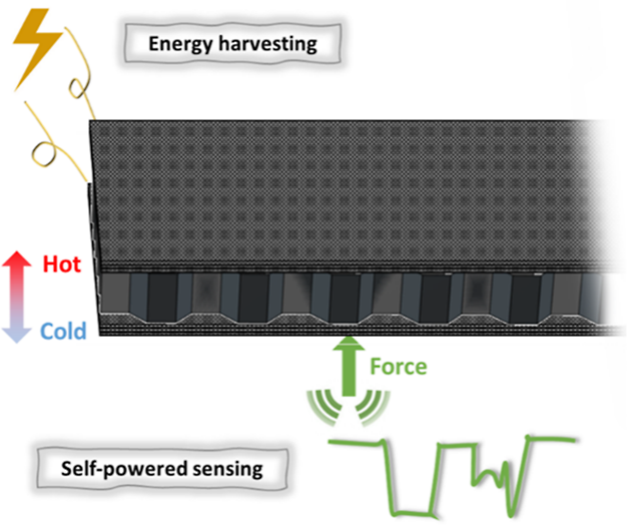

The development of high-performance self-powered sensors in advanced composites addresses the increasing demands of various fields such as aerospace, wearable electronics, healthcare devices, and the Internet-of-Things. Among different energy sources, the thermoelectric (TE) effect which converts ambient temperature gradients to electric energy is of particular interest. However, challenges remain on how to increase the power output as well as how to harvest thermal energy at the out-of-plane direction in high-performance fiber-reinforced composite laminates, greatly limiting the pace of advance in this evolving field. Herein, we utilize a temperature-induced self-folding process together with continuous carbon nanotube veils to overcome these two challenges simultaneously, achieving a high TE output (21 mV and 812 nW at a temperature difference of 17 °C only) in structural composites with the capability to harvest the thermal energy from out-of-plane direction. Real-time self-powered deformation and damage sensing is achieved in fabricated composite laminates based on a thermal gradient of 17 °C only, without the need of any external power supply, opening up new areas of autonomous self-powered sensing in high-performance applications based on TE materials.

## 1 Introduction

Thermoelectric (TE) modules consist of alternating p–n legs that can convert thermal energy from ambient environments to electric energy, allowing to power up existing modules without needing external power supply or batteries. This is particularly attractive in many fields like wearable electronics, robotics, wireless sensors, and high-performance composite materials, especially those systems in remote or hard-to-access locations where reliance on external batteries should be minimized.

Compared with various organic TE materials such as derives of poly(3,4-ethylenedioxythiophene) (PEDOT) and poly(3-hexylthiophene-2,5-diyl) (P3HT), carbon-based (nano)materials have achieved rapid development with many promising results over past few years due to their relatively low cost and environmental sustainability. In particular, great research interest can be found in carbon nanotube (CNT)-based TE materials thanks to their various self-standing assemblies such as fibers, yarns, veils, and fabrics available in large quantities *via* continuous production,^1,2^ allowing easy integration and fabrication alongside tunable TE properties,^3–5^ high electrical conductivity,^6,7^ and potential mechanical reinforcement.^8^ Ultrahigh power factors of 2482 μW m^–1^ K^–2^^7,9^ and 3103 μW m^–1^ K^–2^^10^ at room temperature have been reported for both single-walled carbon nanotube and multiwalled carbon nanotube continuous films, respectively.

To achieve a high-power output from the TE device, a feasible route to connect p-type and n-type alternating legs is equally important to the high-performance TE material itself, which is sometimes overlooked and hence limits their practical applications. Only few attempts can be found in realizing p–n connection for CNT assembly-based TE devices. Zhou *et al.*(7) reported a compact-configuration flexible TE modules based on the vacuum-filtrated CNT film with a thermopower of 410 μV K^–1^ and maximum 2.51 μW power output achieved at Δ*T* ∼ 27.5 °C at the in-plane direction. Choi *et al.*(11) fabricated a flexible TE generator to harvest the thermal energy in the out-of-plane direction by doping the continuous CNT yarns with alternating p-type and n-type around a polydimethylsiloxane (PDMS) block, with a maximum power density of 697 μW g^–1^ at Δ*T* ∼ 40 K based on 90 pairs of p–n connections. Choi *et al.*(12) also demonstrated that with nine pairs of p–n connections, the CNT films can generate 3.4 mV by converting body heat directly (Δ*T* ∼ 7 °C).

In fact, only since last year, a few efforts can be found in utilizing integrated TE modules in high-performance lightweight structural applications where many waste heat is available. Paipetis’ group presented a CNT-painted glass fiber with the cost of either need in-plane temperature gradience^13^ or only achieve a single-leg device.^14^ In 2019,^15^ Karalis *et al.* utilized a series of p-type- and n-type-doped commercial carbon fiber tows as a bottom ply of a structural TE composite with five pairs of p–n legs generating 19.56 mV voltage output at Δ*T* ∼ 75K from in-plane thermal gradient. Very recently, Karalis *et al.* used CNT inks to coat on glass fiber fabrics and built eight pairs of p–n layers alternating between insulating glass fibers, achieving a power output of 2.2 μW at Δ*T* of 100 K from in-plane thermal gradient. Although the feasibility of integrating TE modules into structural composites has been successfully demonstrated, it is worth noting that many of the thermal gradients in fiber-reinforced composite applications are found at the out-of-plane (through thickness) direction.

Clearly, to utilize the TE effect in nanoengineered high-performance composites, two obvious and practical challenges remain: (i) how to utilize the out-of-plane thermal gradient since most of the thermal gradients exist across the thickness direction of the components rather than in-plane and (ii) how to increase the power output from TE modules, or in other words, how to effectively connects the alternating p–n legs. Another key design criterion for any nanoengineered composites is the integration of the system without significantly affecting the original performance while adding new functionalities.

Herein, we present an innovative strategy to achieve high-power output TE in structural composites, addressing all three challenges simultaneously by utilizing a Kirigami-inspired self-folding process to establish a CNT film-based honeycomb structure. The well-acknowledged mechanically robust honeycomb structure can enable not only the utilization of thermal gradient along the out-of-plane direction but also the capability for alternating p–n legs to be well connected for energy harvesting. The electrical power output obtained from this integrated TE honeycomb in hierarchical composites is sufficient to perform the *in situ* deformation and damage sensing, providing added multifunctionalities such as self-powered structural health monitoring. This new method to integrate TE module into hierarchical composites *via* self-folding to form a honeycomb structure could be used in various high-performance composite applications in the fields of aerospace, automotive, and renewable (solar) energy sectors, especially at remote and hard-to-access locations.

## 2 Materials and Methods

### 2.1 CNT Veil Fabrication

CNT veils were made by the floating catalyst chemical vapor deposition (FCCVD) method. The feedstock consists of about 96.5 wt % ethanol (carbon source), 1.9 wt % ferrocene (catalyst precursor), and 1.6 wt % thiophene (promoter) and was injected (at a rate of 0.15 mL/min) into a CVD furnace (∼1150 °C) along with the carrier gas (at a rate of 600 mL/min) of hydrogen and argon (*ca.* 1:1 in volume). Detailed fabrication procedures can be found in previous publications.^16,17^ The CNTs were formed and entangled into a sock-like aerogel in the furnace, then was pulled out, and collected by a rotating roller continuously. As a result, the high-porosity CNT sponge on the roller was half-densified by mechanical compression, followed by the annealing-acid wash procedure.

The “as-grown” CNT for veils has been annealed at 450 °C in air for 1 h and then immersed in hydrochloric acid (36–38%) for 12 h. Afterward, the CNT veils have been washed in deionized water several times until the pH value reaches 6–7 and then dried in the oven at 100 °C for 2 h before testing and doping.

Polyethylenimine (PEI) and FeCl_3_ were dissolved in ethanol and used for n- and p-type doping, respectively. Different concentrations of the dopant solutions have been used ranging from 2 to 20 mM. For comparison, the amount of the dopant solutions is all kept at 50 μL and dropped onto a 15 mm × 15 mm squared CNT veil on glass slide substrates.

### 2.2 Self-Folding TE Module Structures and Fabrication

A 250 μm-thick polycarbonate (PC) film (LEXAN 8010 Film) was provided by SABIC. Three types of PC patches, small (8 mm × 1.5 mm), medium (8 mm × 8.5 mm), and large (8 mm × 10 mm), have been cut by Silhouette cameo. A commercial bioriented polystyrene (b-PS) film (Grafix shrink film) was cut into the designed patterns. CNT veils were cut into the same pattern as b-PS and then densified by a few drops of ethanol in order to adhere CNT onto both sides of the b-PS film. After the doped CNT veils being dried at 40 °C, cyanoacrylate glue (Loctite, Henkel Ltd.) was used to adhesively bond the CNT veils onto the b-PS substrate and PC patches. The as-assembled sample was then dried at room temperature overnight, before placing into the 130 °C oven for self-folding processes.

### 2.3 Self-Powered Nanoengineered Composite Laminates

A total of 60 mg of carbon nanotubes (NC7000, Nanocyl S.A.) was dispersed in acetone by probe sonication with 5000 J energy at 20% of the maximum amplitude level. The spray coating was performed using an airbrush (H4001 HP-CPLUS, Iwata Performance) connected to the air compressor (Iwata studio series) to deposit CNTs onto the surface of a 10 × 10 cm twill glass fiber/epoxy prepreg (MTC510 from SHD Composites). It is worth mentioning that an electrically insulating glass fiber reinforcement has been chosen for this work in order to avoid the electrical short connections between the plies. 30 psi (2.07 bar) air pressure and a 10 cm distance between the spraying nozzle and prepreg were used as mentioned in our previous study.^18,19^ The measured 60 mg of CNTs was spray-coated onto the top surface of prepreg, resulting in ∼7 wt % CNT loading to the resin. The CNT-coated prepreg was then placed on top of another four piles of uncoated prepregs, with the coated surface facing up as the outer conductive layer. After degassed under vacuum for 30 min to avoid any trapped air, a curing cycle of 120 °C for 2 h with a heating rate of 3 °C/min from room temperature was employed with an active vacuum applied throughout. Thin copper wires were used as electrodes to connect the TE module and sensing surface of the fabricated composite laminates.

### 2.4 Finite Element Method Modeling

A multiphysics finite element method (FEM) model in Abaqus was used to model the self-folding process. A first transient heat-transfer simulation to reproduce the composite heating was used as the input for the thermal properties of the materials and the external temperature. The efficiency of the heat transfer has been adjusted to match the experimental results. The dynamic model for reproducing the self-folding behavior of the structure is based on an explicit formulation with the input of the simulated temperature profile. The temperature profile is assumed to be homogeneous inside of the geometry due to its low thickness. The large strains taking place in the active material due to the temperature change led to mesh distortion issues, so an adaptive mesh and a fine discretization in time are required.

### 2.5 Characterization

A two-probe method has been used to measure and compare the resistance change before and after folding for CNT specimens. A bespoke four-point probe system consisting of an Agilent 6614 System DC power supply, a Keithley 6485 picometer, and a Keithley 2000 multimeter was used for electrical conductivity of the as-fabricated and doped CNT veils with a probe space 0.25 mm. The thickness of the as-fabricated and doped CNT veils was measured by a Bruker Dektak Vision 64 profilometer. The Seebeck coefficient was measured at 27 °C under a nitrogen atmosphere using the MMR Technology Seebeck Effect Measurement System.

The folding angle of the samples was recorded by taking the live videos from the side views at 120 °C and then analyzed by ImageJ software. The infrared camera (FLIR E40) was used to monitor the temperature gradient between two sides of laminates, with temperature analyzed by the FLIR software. Scanning electron microscopy (SEM, FEI Inspect F) was used to examine the morphology of samples, with 3 kV accelerating voltage used. The thermal stability was characterized through thermogravimetric analysis (TA Instruments Q500) with applied temperature from 20 to 900 °C at a rate of 10 °C min^–1^ in air, with isothermal steps of 10 min at both the start and final temperatures. The Raman spectra were collected from an inVia Qontor confocal Raman microscope with a 633 nm laser source for 5% power under 50× magnification.

Subjected to a given temperature gradient, the open-circuit voltage of the self-folded module was measured directly by a Keithley 2000 multimeter. For the power output measurements, a variable resistor was connected to the self-folded TE module. Voltage and current were recorded simultaneously by a voltmeter (Keithley 2000 Multimeter) and a picoammeter (Keithley 6485) with the resistor change from 0 Ω to 999 MΩ. The maximum power output can be obtained when the resistance of the load equals to the inner resistance of the module.

For the *in situ* damage sensing tests, the electrical resistance change of the composite panel was recorded by an Agilent 34401A 61/2 digital multimeter during the three-point bending tests. A picoammeter (Keithley 6485) was used to record the current output generated from the honeycomb TE module in real time under a temperature difference of 17 °C for the self-powered sensing setup. Silver paints were used to eliminate the contact resistance between specimens and electrodes. All three-point bending tests were performed in accordance with ASTM D790, with the sample dimensions of 12.7 mm × 85 mm × 3 mm.

## 3 Results and Discussion

### 3.1 TE Performance of CNT Films

The TE properties of CNT films have been systematically characterized, with annealing and purification processes employed to improve their performance. The effect of subsequent folding steps on the electrical properties has also been examined. A large-sized CNT veil (1.5 m^2^) has been manufactured by a FCCVD method. Since the TE property of CNT veils can be affected by the residual impurities such as metal catalysts and amorphous carbon from the manufacturing processes, an annealing process at 450 °C in air environments has also been employed in order to remove organic impurities.

**Figure 1:**
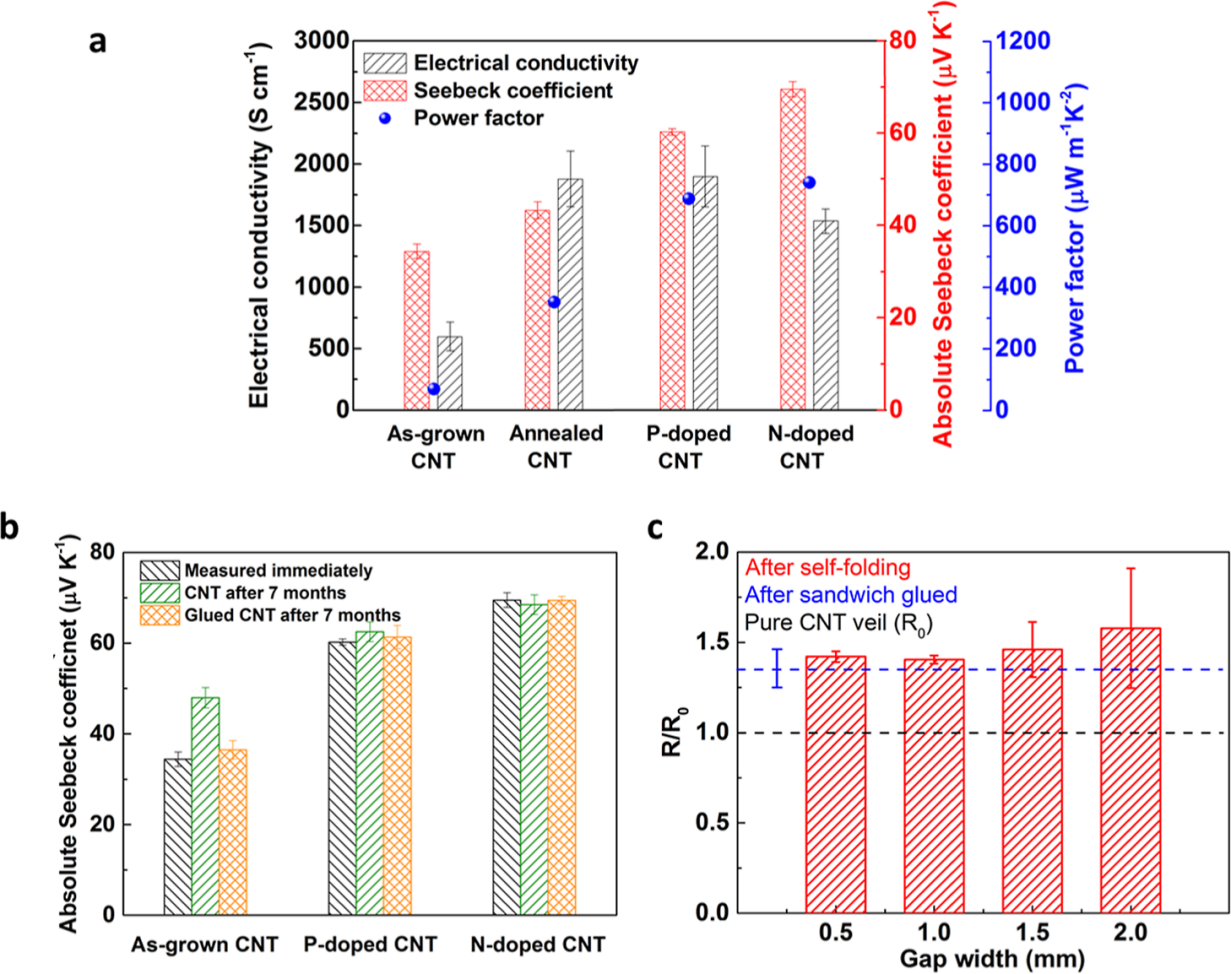
TE properties of CNT veils. (a) Electrical conductivities, Seebeck coefficients, and power factor of the as-grown, annealed, purified (acid-washed after annealed), and p- and n-doped CNT veils at room temperature, with improved properties after annealing processes. (b) Seebeck coefficients of as-grown, p- and n-doped CNT veils change in 7 months and the effect of the glue, indicating a stable TE performance from current CNT films. (c) Effect of glue and folding processes on the electrical resistance of CNT veils, showing a slightly increased resistance value after applying glue and subsequent folding.

Both the electrical conductivity and Seebeck coefficient (Figure 1a) have been significantly improved by applied annealing processes. The electrical conductivity tripled from 598 S cm^–1^ to 1878 S cm^–1^ and the Seebeck coefficient increased from 34 to 42 μV K^–1^. This is attributed to the successful removal of the amorphous carbon by the annealing processes. It is well acknowledged that CNT veils can absorb oxygen and/or moisture from the environments, resulting in the formation of hole-like carriers acting as p-type dopants,^20^ hence an increasing trend of p-type TE property after several days of exposure to the environments. For example, the as-grown CNT veil’s Seebeck coefficient increases from 34 to 47 μV K^–1^ (Figure S1) exposed in air (1 atm, 25–27 °C, and relative humidity 65%) for 200 days. However, this inflated value is unstable and varies depending on the environment. For example, with the high temperature temporarily absorbed oxygen and/or moisture being removed, its Seebeck coefficient will reduce back (Figure S2). Therefore, p-type doping is still necessary to maintain the CNT veil a stable Seebeck coefficient and power factor under different situations. In the meantime, the Seebeck coefficient values in Figure 1a,b were measured after at least 72 h of the treatment and under vacuum for 30 min to ensure consistent and reliable results, eliminating these potential influences.

Although an additional step of purification by acid washing can further improve the TE performance of CNT veils (the Seebeck coefficient further increases to 60 μV K^–1^ with a power factor reaching 1000 μW m^–1^K^–2^), the condensed veil network has inevitably impeded the subsequent doping process (Supporting Information Sections 1 and 2). Therefore, the doping of annealed CNT veils (without further acid washing) was successfully achieved by using FeCl_3_ and PEI, with an optimized Seebeck coefficient of 60 μV K^–1^ for p-type and −70 μV K^–1^ for n-type, respectively. Their electrical conductivity remained at the same level after the doping process, leading to an enhanced power factor of 688 and 741 μW m^–1^ K^–2^ for p-type and n-type at room temperature, respectively (Figure 1a). The TE property is comparable to the literature as summarized in Table 1. As shown in Figure 1b, the stability of Seebeck coefficient has also been improved after performing the doping process, with no obvious changes after 7 months in normal environments.

**Table 1:** TE Property Summary of the CNT-Based Materials and Corresponding TE Generators

materials	σ (S cm^–1^)	α (μV K^–1^)	PF (μW m^1–^ K^–2^)	ref
this work	1878; 1500	60; −70	688; 741	
doped CNT fibers wrapped with acrylic fibers	950	–64	330	(21)
CNT fiber	3000	90	2500	(9)
Au-doped CNT webs	1090–5134	80	3548	(22)
NaBH_4_-doped CNT film	2000	–80	1280	(23)
PEI-doped CNT	600	–58	201	(5)
CNT with PEDOT:PSS	1300	–35	160	(24)
CNT yarns	3147	50; −31	160	(11)
single-walled CNT buckypapers	600	–38	90	(26)
carbon fiber composites	1630	33.85; −11.83	186; 22.8	(15)
PEDOT:PSS/CNT composite yarn	1043	70.1	512.8	(27)
CNT fiber	1899	26.7	432	(28)
CNT fiber	52	–86	40	(29)
CNT fabric	200	47.8; −49.1	40	(30)

To ensure a consistent and reliable electrical and TE performance of the CNT veils after subsequent fabrication and deformation in structural composites, the effect of deformation (folding) and employed adhesive in subsequent processes has also been examined (Figure 1b,c). As expected, with the electrically insulating cyanoacrylate used as glue, CNT composites have shown a ∼ 30% resistance increase compared with the pristine CNT veils of the same dimensions (blue dashed line in Figure 1c). However, no change in the Seebeck coefficient values was found (Figure 1b). It is also worth noting that the insulating cyanoacrylate encapsulated the CNT veils to further avoid the oxygen absorption, ensuring a stable Seebeck coefficient after a long period of exposure (7 months) for the long-term stability and reliability of the fabricated devices. Figure 1c shows the increase in electrical resistance due to the deformation of CNT veils from flat (0°) to a small angle (150–180°) during the folding process, with only limited changes in resistance values from encapsulated CNT specimens regardless of the gap width in folding structures.

### 3.2 Design and Fabrication of the Engineered Modular Structure for CNT Honeycombs *via* Temperature-Induced Self-Folding Process

Although the honeycomb structure is well developed as the core layer for sandwich structural applications, the folding process to turn the two-dimensional (2D) flat CNT veils into three-dimensional (3D) honeycomb modules with accurately connected alternating p–n legs remains a complex and challenging task. As mentioned earlier, a Kirigami-inspired self-folding process is utilized in this work to achieve a honeycomb-structured TE module which can harvest the thermal energy from the out-of-plate direction with connected p–n legs.

**Figure 2:**
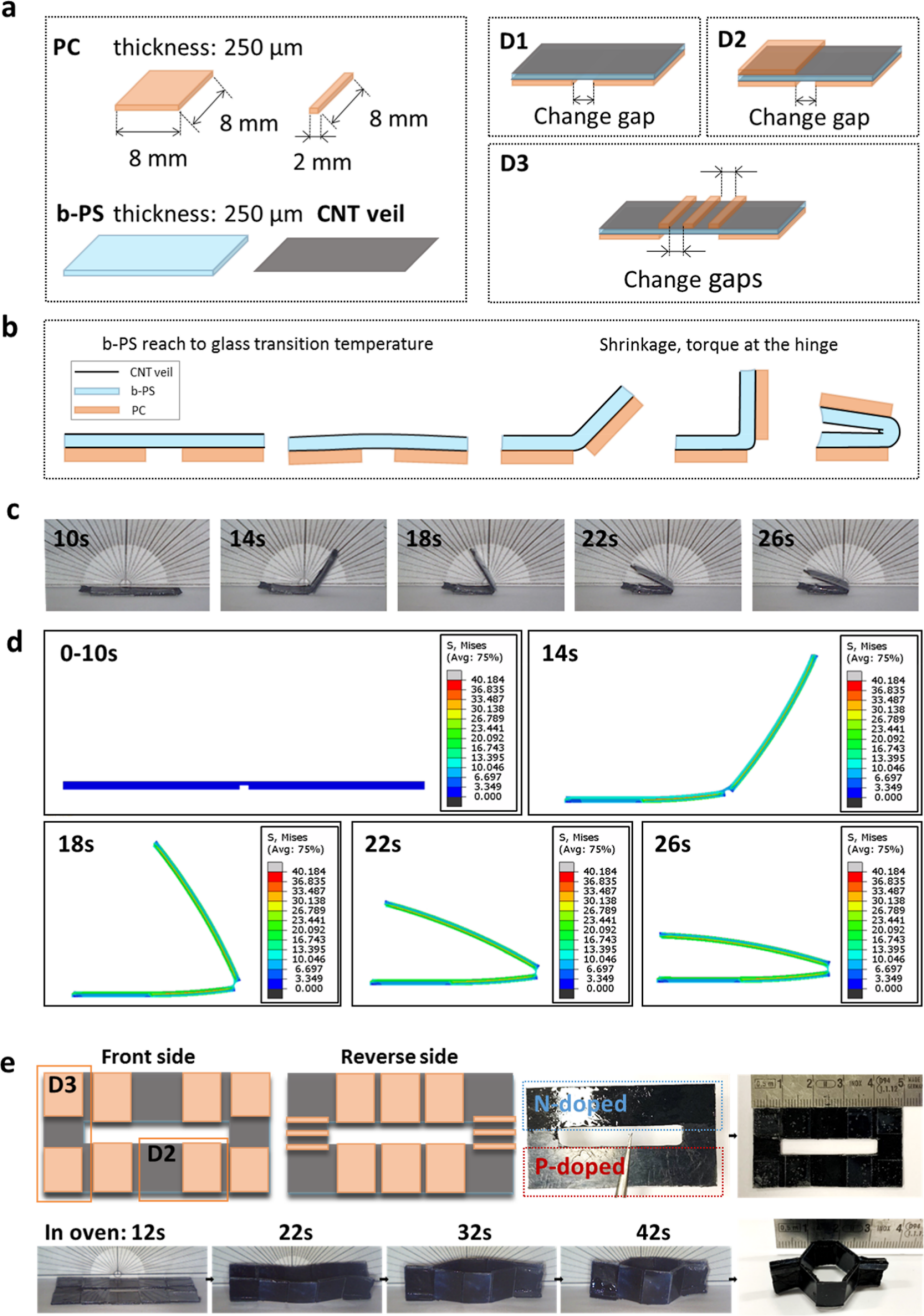
Engineered modular structure of self-folded honeycomb *via* temperature-induced self-folding processes. (a) Schematic illustrations of three structure designs (D1–3) assembled by CNT veils, b-PS, and PC. (b) Schematic illustrations of the self-folding process for D1. (c) Images taken at different times of the self-folding process of D1 with a gap width of 1 mm, showing the folding angles as the function of time at 130 °C. (d) Finite element method analysis of design D1 with the local stress values. (e) Designs and patterns for the modular unit cell honeycomb structure TE module, with circled areas of D2 and D3; and the images of self-folding processes at different stages.

Inspired by our recent work,^31^ a bistretched polystyrene film (b-PS) was used as an active layer with the capability to shrink upon heating, together with a PC layer adhered on top acting as substrates and hinges to restrict the thermal shrinkage, hence achieving the temperature-induced folding process. As shown in Figure 2a, two thin layers of CNT veils (3–5 μm individually) were used to sandwich the shrinkable b-PS layer adhesively, with the PC layer adhered at the outside (either top or bottom) of CNT/b-PS/CNT structures. By changing the location and patterns of an intact PC layer, various shapes and dimensions can be programmed and achieved by the temperature-induced self-folding processes. In order to understand the self-folding mechanism, hence utilizing this method to turn 1D CNT veils into a 3D honeycomb structure with an accurate p–n leg connection, different designs have been examined as well (D1–3 in Figure 2a). To establish the folding profile and relationship between folding angles with time and temperature, a simple design (D1), as illustrated in Figure 2b, has been employed (Figures 2c and S8). Upon increased temperature in the first 10s, the multilayered design D1 first went through a slight opening of the hinge (up to −5°). As the sample was reaching the glass-transition temperature of PS (around 110 °C as shown in Figure S8a), a minor expansion of PS (about 0.1%) occurred. Then the sample started folding at time ≈12s, until reaching a maximum point. At this stage, the release of local stresses of the oriented PS molecular chains led to the contraction of b-PS layer (max. 50%), while the PC layers remained intact and constrained the shrinkage of the PS layer, creating the torque at the hinges and generating the bending moment for the folding process. Very short response time (between 10 and 30 s) was required for this temperature-induced self-folding process, with a great programmability in both folding angles (ranging from 150 to 180°) and folding speed by tuning the gap width between intact layers (Figure S8b). This folding process has also been simulated by the FEM modeling (Figure 2d and Video S1) and fits well with the experimental results (Figure S9). After optimizing the gap width and designs with the aim of achieving the correct angles required for honeycomb structures (Figure S8c,d), D2 with a gap width of 0.5 mm and D3 with a gap width of 1 mm were employed to create the folding angle of 60 and 180° for honeycomb structures, respectively. As shown in Figure 2e, the p and n doping has been employed prior to the folding process, with the patterns suit the honeycomb shape with p–n legs connected autonomously for TE energy harvesting. The single modular honeycomb structure consists of one unit cell can be self-folded at 130 °C within a minute, with the sequential folding of D3 followed by D2 as demonstrated in Figure 2e.

**Figure 3:**
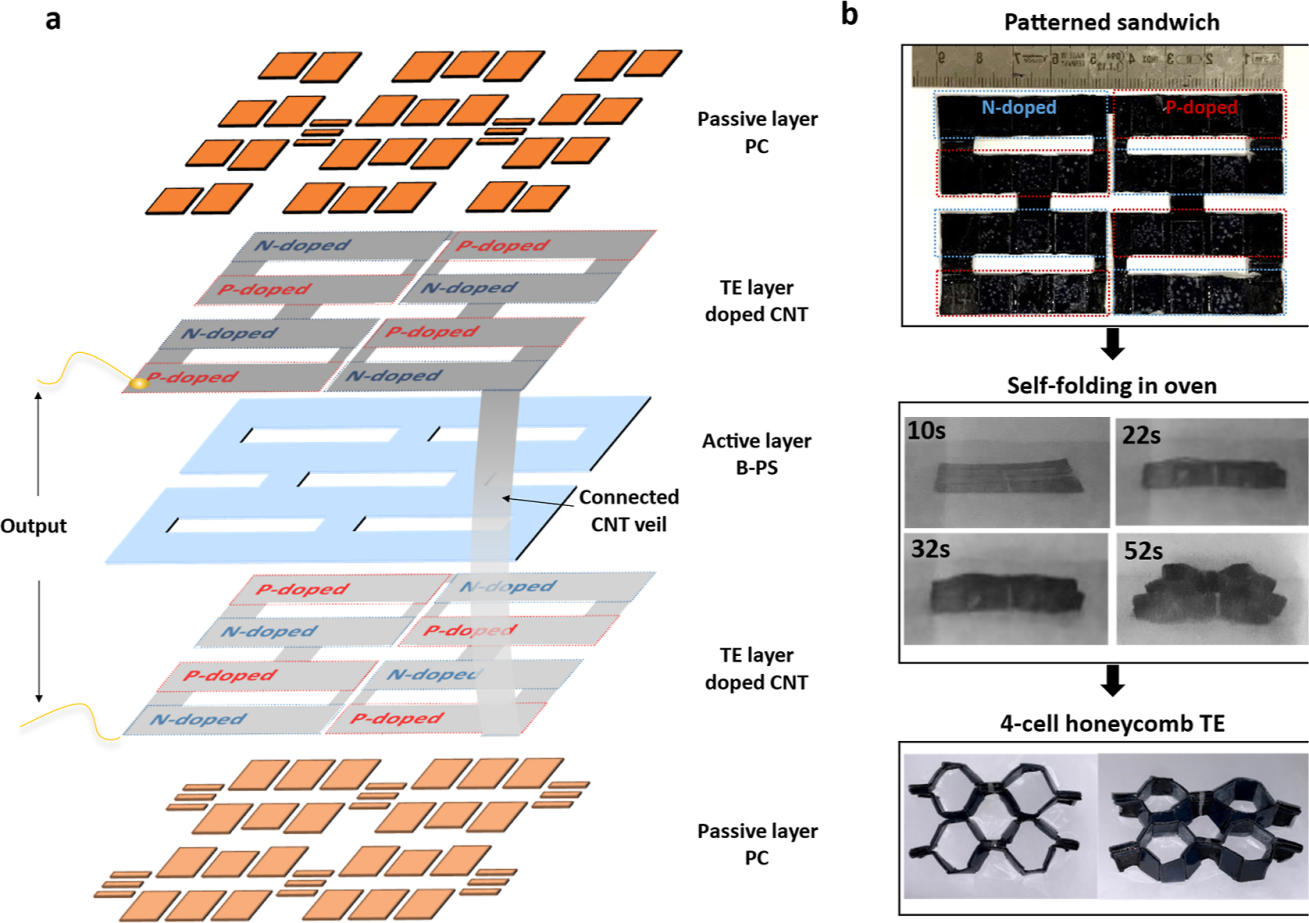
Engineered modular structure of a self-folded four-unit cell honeycomb. (a) Exploded view of the sandwiched honeycomb pattern composed of D2- and D3-patterned PC as a passive layer, b-PS as the middle active layer, and tailored p&n-type-doped CNT veils as an interlayer for TE property. (b) Images of the fabrication process of the TE module with a four-unit cell honeycomb structure *via* self-folding processes.

To demonstrate the feasibility of utilizing this self-folding process for high TE power output, a honeycomb structure consisting of four-unit cells has been fabricated. Benefitted from the formation of four pairs of alternating p–n legs on each side of the patterned b-PS layer, eight thermocouples have been achieved in this self-folded four-cell honeycomb TE module (Figures 3 and S10). Similar to the doping pattern of the single module, the undoped regions (Figure 3a, connected CNT veil) were folded to both sides of the module and acting as the electrodes to minimize the internal resistance of the fabricated TE module. The resistance of a four-unit cell TE module is 12 Ω, which can be attributed to the continuous CNT veils with tailored patterns. Clearly, the vertical alignment of alternating p–n CNT legs in these fabricated TE modules can enable the thermal energy harvesting from the out-of-plane thermal gradients autonomously, opening up a much wider field of practical applications.

### 3.3 TE Performance of Self-Folded CNT Honeycomb Modular Structures

**Figure 4:**
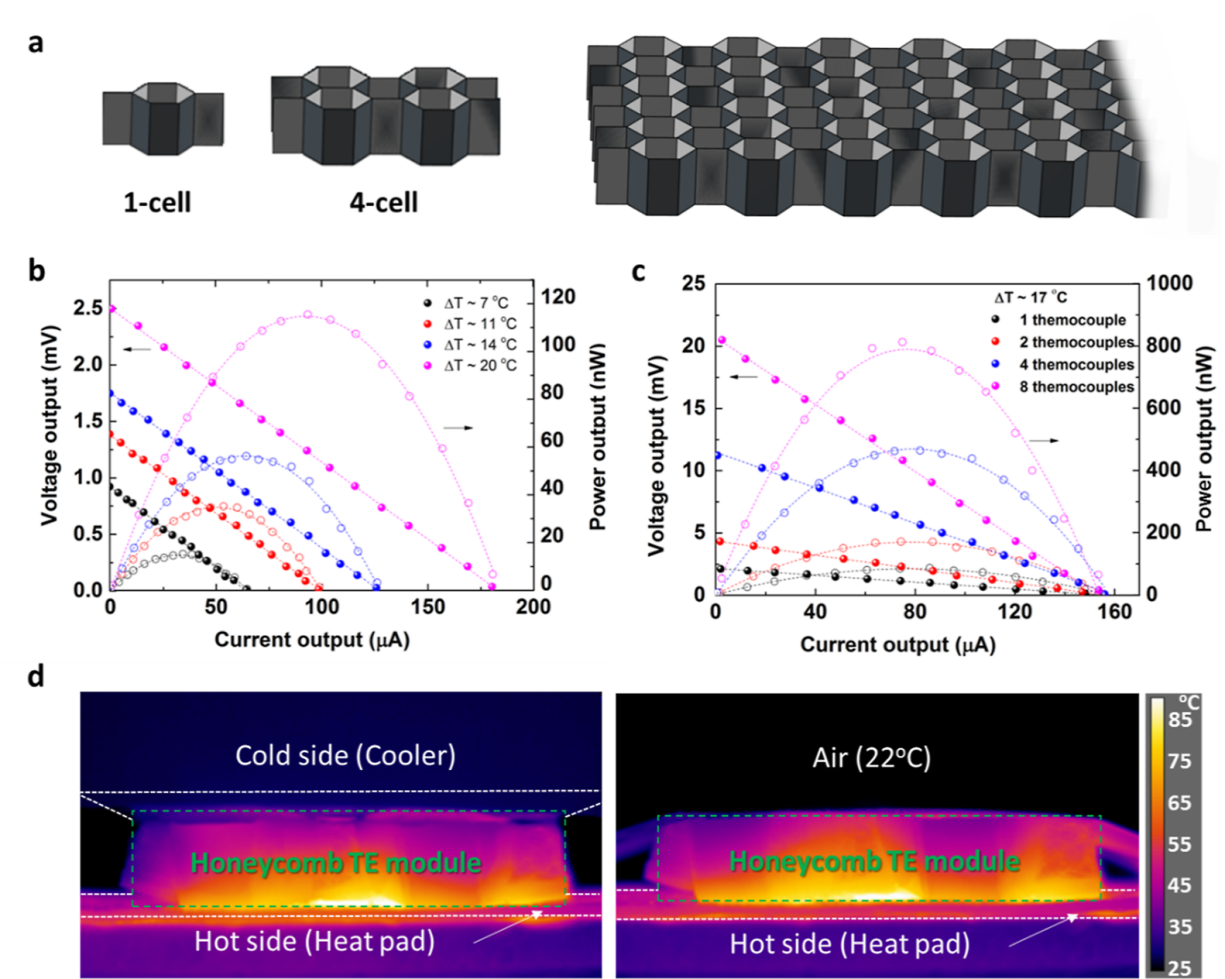
TE performance of CNT honeycomb structures. (a) Schematic illustrations of the self-folded single-unit cell, four-cell, and multicell honeycomb-structured TE modules; (b) voltage and power output from a single-unit cell honeycomb TE module that consists of a single thermal couple at various temperature differences, with peak outputs of 2.5 mV and 115 nW at Δ*T* ∼ 20 °C; (c) voltage and power output from different numbers of thermocouples, with peak outputs of 21 mV and 812 nW from the four-unit cell (eight thermocouples) honeycomb TE module at Δ*T* ∼ 17 °C; and (d) thermal images of the cross-sectional views of the four-unit cell honeycomb TE module, confirming a stable thermal gradient regardless of an active cooling at the top surface.

To evaluate the TE performance and power output of the fabricated CNT honeycombs, the single-unit cell consists of one pair of p–n legs has been examined under various temperature differences at the out-of-plane direction (Figure 4a). Under a Δ*T* ∼ 20 °C, a single pair of the p–n legs can generate a voltage of 2.5 mV with a peak power output up to 115 nW (Figure 4b), which is already higher than most reported values from CNT-based TE modules in the literature.^3,32^ Theoretically, an increasing number of the thermocouples (*n*) can increase both the voltage output *U* and the maximum power output *P*_max_. Therefore, the energy harvesting performance of the four-unit cell TE module has also been examined to demonstrate the potential of further enhancing the output power by increasing the number of thermocouples in series. As shown in the following equations, the maximum power output should be obtained when the externally loaded resistance is equal to the sum of internal resistance (*R*_i_) of the TE module and contact resistance (*R*_c_).
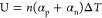
1
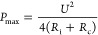
2where α_p_ and α_n_ refer to the absolute values of the Seebeck coefficient of the p- and n-doped CNT veils, which are 60 and 69 μV K^–1^, respectively. Therefore, under a constant temperature difference (Δ*T*), *U* ∝ *n*. Meanwhile, the internal resistance is also increasing linearly with the number of thermocouples due to the increased number of series connected of CNT electrodes. Thus, for a TE module with a large number of the thermocouples, R_i_ should be much higher than *R*_c_ and increases linearly with the number of thermocouples since the average resistance per p–n pair is nearly constant, while *P*_max_ should be proportional to *n*.

The measured power output in Figure 4c is in good agreement with this relationship. The highest voltage generated by the four-cell honeycomb structure with eight thermocouples was around 21 mV, together with a maximum power output of 812 nW under 17 °C temperature difference, exceeding the reported values in composites from the literature with a much lower temperature difference.^15^ These values are around 8 times of the energy harvesting capability from the single-unit thermocouples, which are in consistent with the reported literature in boosting power output by increasing numbers of paired p–n legs.^33^ The output power density of the four-unit cell TE device is 6940 mW g^–1^ (for pure CNT veils without polymer layers) at per temperature difference squared Δ*T*^2^, with detailed calculation show in Supporting Information Section 3 and comparison in Table 2. Obviously, both the voltage output and power output can be further increased with increasing numbers of unit cells in the current honeycomb-structured TE module. Based on the current relationship between numbers of connected thermocouples and obtained power output, 10 μW can be achieved in the honeycomb TE module consisting of a 50-unit cell (100 effective thermocouples) at a temperature difference of 17 °C only, fulfilling the practical requirements of many electronics and devices. Besides, the durability of the self-folded TE module after more than 3 years under atmosphere is also reported in Figure S12. The four-thermocouple-structured TE module still can provide 4 mV voltage output with a peak power output of 40 nW under 14 °C temperature difference.

**Table 2:** TE Property Summary of the CNT-Based TE Generators

device	p&n pairs	power output (μW m^–1^ K^–2^)	power density normalized by area (μW m^–2^ K^–2^)	power density normalized by weight (μW g^–1^ K^–2^)	ref
this work	8	21 mV and 812 nW at Δ*T* ∼ 17 K	2.82	6940 (CNT veils only) 0.0115 (four-cell device with polymer layers)^a^	
doped CNT fibers wrapped with acrylic fibers	15	70 mW m^–2^ at Δ*T* ∼ 44 K	35		(21)
Au-doped CNT webs	7	1.74 μW at Δ*T* ∼ 20 K	10	1.4	(22)
NaBH_4_-doped CNT film	1	6 mV, 25 nW at Δ*T*∼ 22K	0.0001		(23)
CNT yarns	60	10.85 μW g^–1^ at d*T*∼ 5 K 697 μW g^–1^ at d*T* ∼ 40 K		2.17	(11)
single-walled CNT buckypapers	37.5	10.3 μW at Δ*T*∼ 30 K	0.15		(26)
carbon fiber composites	10	20 mV, 50 μW at Δ*T*∼ 75 K	0.00025		(15)
PEDOT:PSS/CNT composite yarn	966	171.7 μW/(g·K) at Δ*T* ∼ 47.5 K	22.8	3.6	(27)
CNT fiber	40	15.4 μW g^–1^ at Δ*T* ∼ 5K 259 μW g^–1^ at Δ*T* ∼ 20 K		3.08	(28)
CNT fiber	72	150 mV; 31 μW at Δ*T* ∼ 32 K	0.002		(29)
CNT fibric	10	2.3 mV Δ*T* ∼ 5 K	23		(30)

a The weight of PC and PS films in a four-cell device is estimated to be 0.24 g.

Additionally, the existence of a large number of cavities within the honeycomb structure also brings benefits of a low thermal diffusion coefficient at the out-of-plane direction, maintaining a stable temperature difference without the needs of an external cooling system. As shown in Figure 4d, the temperature gradients across the four-unit cell honeycomb TE module were very stable, regardless of the use of a cooling system on the opposite surface. After reaching the thermal equilibrium of the TE module with a bottom heating pad set at 90 °C, the temperature of the cold side was 47 °C without active cooling, translating to a Δ*T* ∼ 43 °C across the TE module. Compared to the system with the cooling system set at the top (Δ*T* ∼ 50 °C), only 14% temperature loss was observed, thanks to the cavities within these honeycomb structures.

### 3.4 Self-Powered Strain and Damage Sensing in High-Performance Composite Laminates

The fabricated honeycomb TE modules can be integrated into composite laminates, adding multifunctionalities such as *in situ* sensing to the components in two different ways: either as a self-powered sensor by harvesting thermal energy to detect the deformation and damage or as a temperature sensor to monitor the external temperature variations by measuring the power output generated from the TE effect.

**Figure 5:**
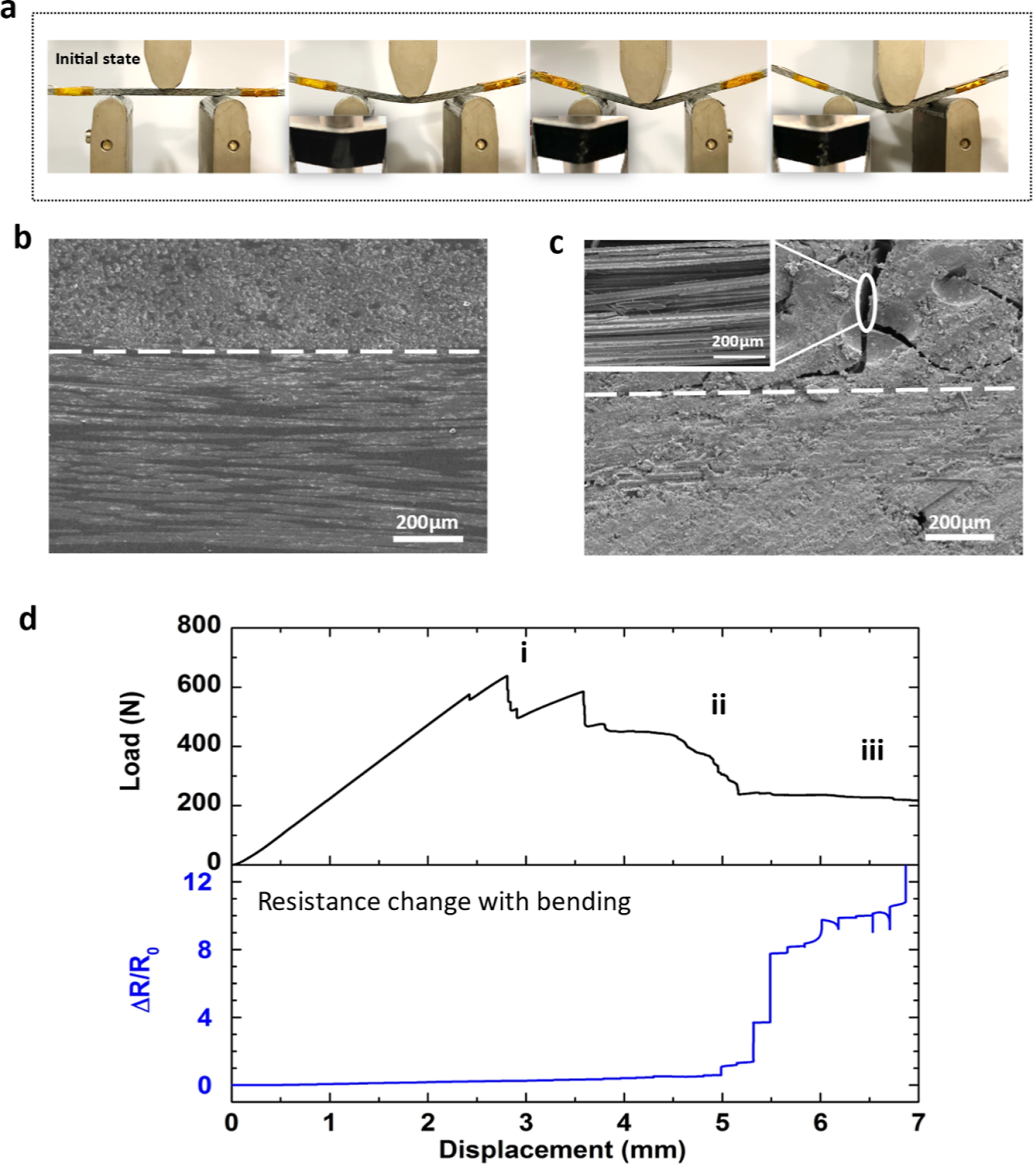
*In situ* sensing in composite laminates under flexural loadings: (a) photos taken from three-point bending tests at different stages of the test; SEM images of the cross-sectional views of the nanoengineered hierarchical composites that consist of glass fiber/epoxy with a thin layer of percolated CNT spray-coated at the top ply (b) before and (c) after the three-point bending test. (d) Electrical resistance sensing based on external power supply, showing clear sensing signals when the damage propagated to the surface sensing layer.

Since the electrical sensing method is utilized for deformation and damage monitoring in multifunctional composites, prior to examining the self-powered sensing capabilities based on the honeycomb TE module, the electrical sensing performance with an external power supply has been evaluated first for the current system. The nanoengineered hierarchical composites consist of glass fiber-reinforced plastics with a thin layer of percolated CNT sensory network spray-coated at the top ply as the sensing layer is used here,^19,34^ with the resistance measured as the sensing signals throughout the flexural tests (Figure 5a). Morphological observations show a good quality of the laminates without any obvious voids (Figure 5b). Upon loading, although matrix cracking and interfacial debonding can be expected at relatively low strains (as evidenced in Figure 5c), the electrical sensing signals remained almost unchanged at the beginning of the test (Figure 5d). This is due to the limited deformation and damage of the sensing layers at the outer layer of the laminates, especially considering the local high CNT loading which is well above the percolation threshold. With the damages accumulated within the laminates and propagated to delamination and fiber breakages, obvious changes in the load curve can be found with clear load drops (annotated as i within Figure 5d), indicating irreversible damages within the specimen. However, due to the relatively high amount of CNTs employed in the sensing layer, no obvious electrical sensing signals can be observed until the cracks have progressed with obvious damage at the outer sensing layer (annotated as ii in Figure 5d), showing obvious jumps in electrical sensing signals. Clear sensing signals can be found with the damage propagating within the sensing layers, with reduced loading levels observed from the load–displacement curve. Although the sensitivity can be adjusted and improved by reducing the amount of CNTs toward the percolation threshold, high initial electrical conductivity might be required for certain applications, therefore at the costs of sensitivity in the current sensing method.

**Figure 6:**
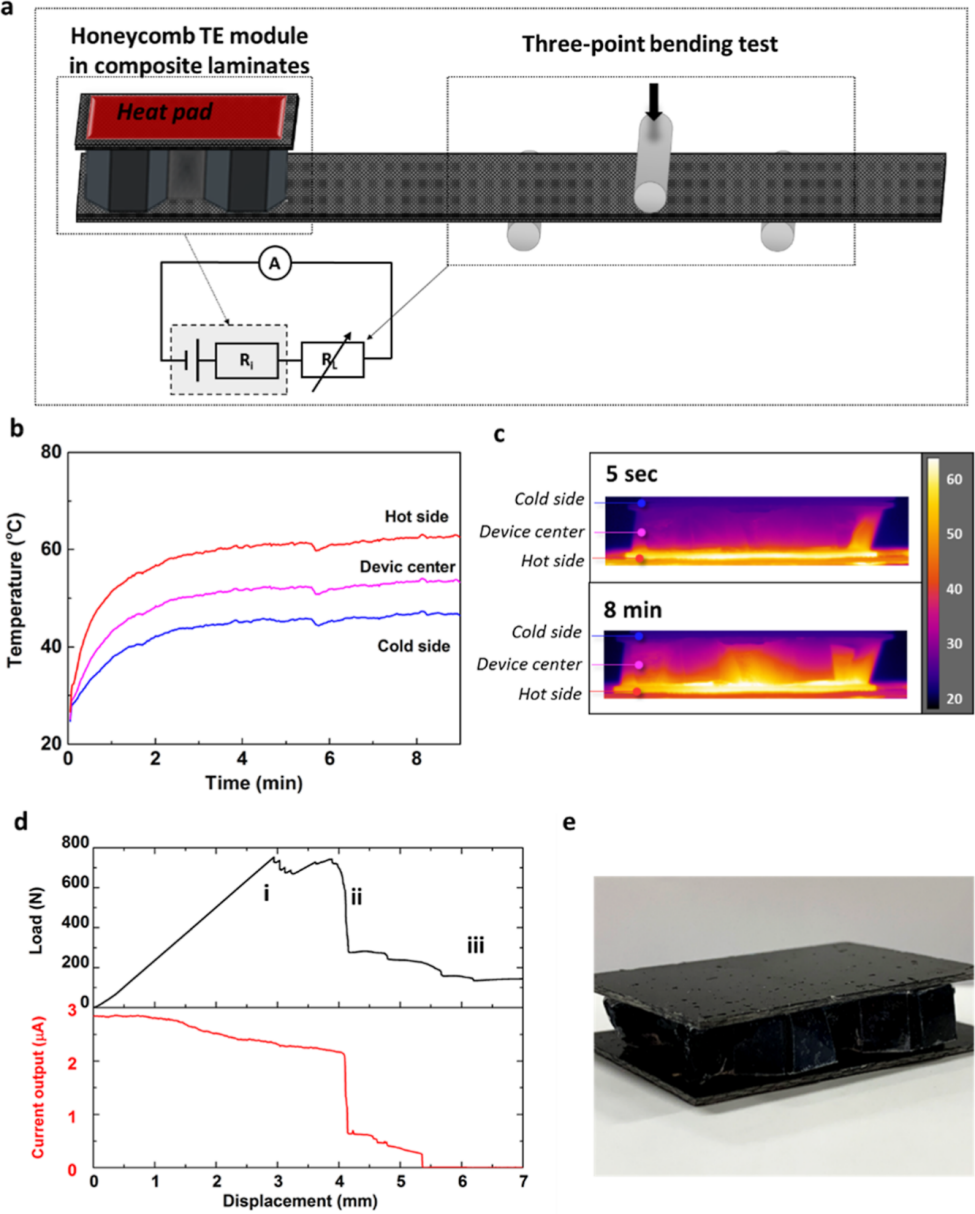
*In situ* self-powered sensing in composite laminates under flexural loadings. (a) Illustration of the current self-powered sensing setup with a honeycomb TE module integrated at the side of the specimen; (b) temperature profiles of the TE module in contact with a 65 °C heat pad in an ambient environment; (c) cross-sectional thermal images of the four-unit cell honeycomb TE module at the beginning and stabilized temperature condition; (d) self-powered sensing based on thermal energy harvesting (17 °C), with a clear signal appeared at a relatively early stage of the test; (e) illustration of an idealized fully optimized sandwiched structure with the CNT honeycomb TE module as the core layer to provide energy harvesting and structural functions.

Instead of relying on external power supply, the current output generated *via* thermal energy harvesting can also be utilized to develop a self-powered sensing system (Figure 6a). The TE module can reach a stable temperature gradient in ∼5 min with a temperature difference of ∼20 °C between top and bottom sides (Figure 6b,c). Under a constant temperature difference, the voltage output generated from the TE module will remain constant. Therefore, any change in electrical resistance from the sensing layer will lead to a change in the current output, which can be utilized as a sensing signal to detect deformation and damage in composite structures.

For the current self-powered sensing method, a stable current output of around 3 μA was achieved with 17 °C of temperature difference (Figure 6d). Upon flexural loading, the electrical sensing signals remained stable at the very beginning and then started to decrease slightly from a relatively early stage of the test (around only 1 mm displacement). A gradual decrease in sensing signal can be found, correlating with increased load and deformation of the specimen. This early detection capability can be attributed to the relatively low absolute value of the current output, leading to a high sensitivity at such low strains. With the load continued to increase, delamination and fiber breakage can be expected with clear load drop from the load–displacement curve (annotated as i in Figure 6d). A further decrease in sensing signals can be found at this stage, regardless of the sensing layer only presented at the outer layer. When the crack propagated and reached the outer sensing layer (annotated as ii in Figure 6c), a very large drop in sensing signals can be found due to the significant change in system resistance value, hence the current output. It is worth noting that no external power supply is required for the honeycomb TE module, while the deformation and health conditions with internal damage propagations can be monitored in real time based on thermal energy harvested in this self-powered sensing method.

Clearly, both external powered sensing and self-powered sensing methods can be utilized to monitor the deformation and damage in real time, while the sensitivity can be adjusted depending on the requirements of applications by utilizing resistance or current changes as sensing signals. As illustrated in Figure 6d, the four-unit cell honeycomb TE module is integrated into the composite laminates but not directly under the flexural loadings during the testing, hence without any effects in mechanical performance. The durability and repeatability of the self-powered sensing property has also been examined with a consistent performance achieved, as shown in Figures S12 and S13. It is worth noting that a fully optimized structure should be developed (Figure 6e) with a tailored honeycomb TE structure. The interfacial adhesion needs to be optimized to fulfill a wider range of applications based on a current self-folded honeycomb in a sandwiched structural laminate. Nevertheless, this has proved the concepts and provided a feasible strategy to scale-up (and scale-down) the TE device by designing and fabricating a patterned passive layers on the active layers, turning a 2D multilayer structure into a 3D energy harvesting device at different length scales. A programmable procedure also allows the sequential self-folding with remote triggering of deployable structures, especially for the applications where space is constrained during the transport stage.

## 4 Conclusions

Self-powered sensing based on thermal energy harvesting has been successfully integrated into a high-performance composite *via* a temperature-induced self-folding process with capability to detect deformation and damage in composite laminates without external power supply. Two long-lasting issues of using thermal electricity in advanced functional composites have been addressed simultaneously, namely, (i) harvesting thermal gradient in the out-of-plane direction and (ii) generating high-power output with a limited temperature difference. High peak outputs of 21 mV and 812 nW at a temperature difference of only 17 °C have been achieved with a CNT honeycomb structure that consists of accurately connected four thermocouples of alternating p–n legs, opening up new routes for self-powered structural health monitoring and thermal energy harvesting in high-performance composite applications.

A temperature induced self-folding process has been utilized in this work, turning 1D continuous CNT veils into 3D honeycomb structures autonomously upon heating. Various designs have been developed and fabricated to achieve both the modular unit of the CNT hexagonal structure as well as the engineered CNT honeycomb structure with accurately connected p–n legs. The TE properties of continuous CNT veils have been systematically examined and tuned for optimized energy output, with limited effects observed from the self-folding process. A linear relationship between the power output and numbers of TE legs under a constant temperature gradient has been validated with the fabricated CNT honeycomb structures, indicating that the power output could reach 10 μW with a 50-unit cell honeycomb at a temperature of 17 °C only, which can fulfill the practical requirements of various electronics and devices. The cavities within the honeycomb structure also enabled low thermal diffusion, hence facilitated a stable temperature gradient across the specimen without the needs of an active cooling system.

Self-powered sensing based on TE effects has been successfully achieved in a structural composite laminate, with energy harvested from the out-of-plane direction without affecting the original performance of the composite laminates. Both elastic deformation and the damage propagation have been monitored in real time, with sensing signals clearly correlated with various stages of damage upon loading, confirming its great potential to be used as a self-powered structural health monitoring system at remote and/or hard-to-access locations for advanced composite applications.

## Supporting Information Available

Folding process (MOV)
Effect of exposure time on the Seebeck coefficient of CNT veils; CNT purification procedures; TGA results, TE properties, and normalized Raman spectra of the as-grown, annealed, and purified CNT; SEM images of CNT veils; optimization procedures and obtained TE properties of doped CNT veils; SEM images of doped CTN veils; self-folding angle with various gap width; comparison between simulation and experimental result on the temperature-induced self-folding process; schematic illustrations of the design for a four-cell honeycomb structure TE module; normalized power output calculation; TE performance of the CNT honeycomb structure after 3.5 years; repeated self-powered sensing results (PDF)

## Supplementary Material

am3c08360_si_001.mov
