# Supplementary material for: Toward Self-Powered Sensing and Thermal Energy Harvesting in High-Performance Composites via Self-Folded Carbon Nanotube Honeycomb Structures
Source: ACS Appl Mater Interfaces. 2023 Sep 11;15(37):44212–23. doi: 10.1021/acsami.3c08360 (PMC10520910; doi:10.1021/acsami.3c08360)
Supplement: Supplementary file 2 — am3c08360_si_002.pdf [file am3c08360_si_002.pdf]

SUPPORTING INFORMATION

Towards self-powered sensing and thermal energy harvesting in high-performance composites via self-folded CNT honeycomb structures

Kening Wan,[a] Arnaud Kernin,[a] Leonardo Ventura,[a] Chongyang Zeng,[a] Yushen Wang,[a] Yi Liu,[a, b], Juan J. Vilatela,[c] Weibang Lu,[d] Emiliano Bilotti,[a,] * Han Zhang[a,*]*

a. School of Engineering and Materials Science, Queen Mary University of London, Mile End Road, London E1 4NS, UK

b. Department of Materials, Loughborough University, Loughborough LE11 3TU, UK

c. IMDEA Materials Institute, Eric Kandel 2, 28906, Getafe, Madrid, Spain

d. Division of Advanced Nanomaterials and Innovation Center for Advanced Nanocomposites, Suzhou Institute of Nano-Tech and Nano-Bionics, Chinese Academy of Sciences, 215123, PR China

* Corresponding authors: han.zhang@qmul.ac.uk; e.bilotti@imperial.ac.uk

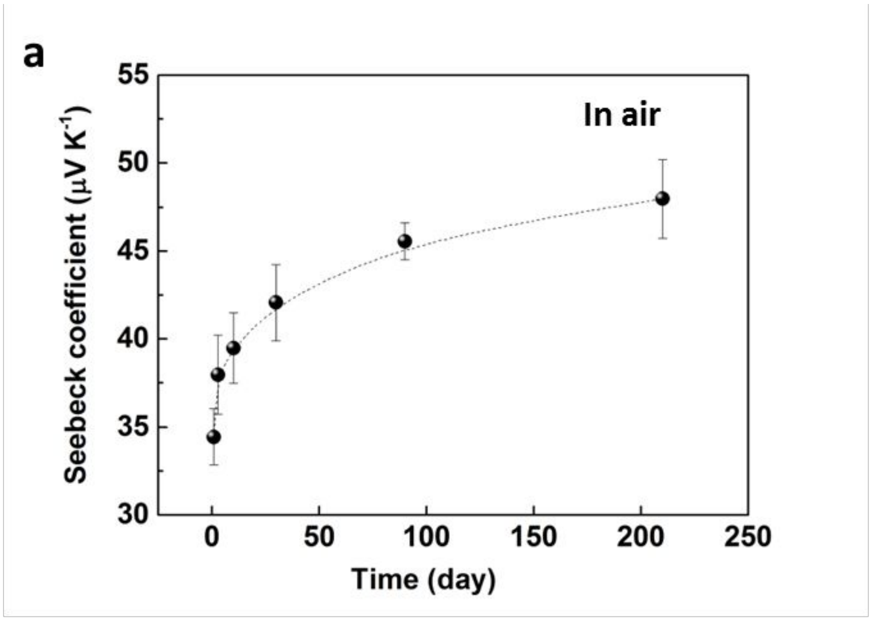

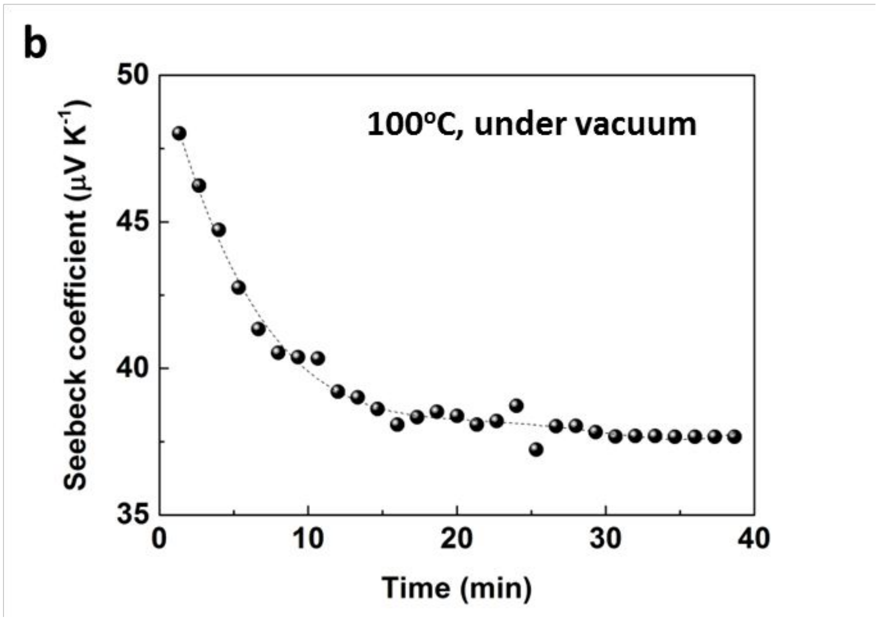

Figure S1. The effect of exposure time on the Seebeck coefficient of CNT veils. (a) Seebeck coefficient of the as-grown CNT veils changes with the function of time exposure in air with 1 atm, 25–27 ˚C and relative humidity of 65%. (b) Seebeck coefficient of the as-grown CNT veils changes with the function of time under 100°C and vacuum.

Section 1 CNT purification

After anealling, a further step of acid washing can remove the residual metal catalysts. Fe content in the purified CNT veil was reduced from 11.5 wt.% to 2.3 wt.% as shown from the TGA results in **Figure S2**. After the acid washing, both the electrical conductivity and Seeback coefficient were further increased to 2315 S cm^{-1} and 65 μV K^{-1} (**Figure S3**), respectively. It is worth noting that this further purification procedure might introduce limited level of defects into CNTs, which was evidenced by the Raman spectroscopy with I_D/I_G ratios increased from 0.19 to 0.30 (**Figure S4**). With the obtained thermopower and electrical conductivity after purification process, a significantly enhanced power factors of 1050 μW $m^{-1}K^{-2}$ was achieved compared to only 70 μW $m^{-1}K^{-2}$ of the as-grown CNT veils. However, it was found that the purified CNT veils have shown a highly condensed morphology (**Figure S5**) which can hindered the infiltration process of dopants in subsequent steps.

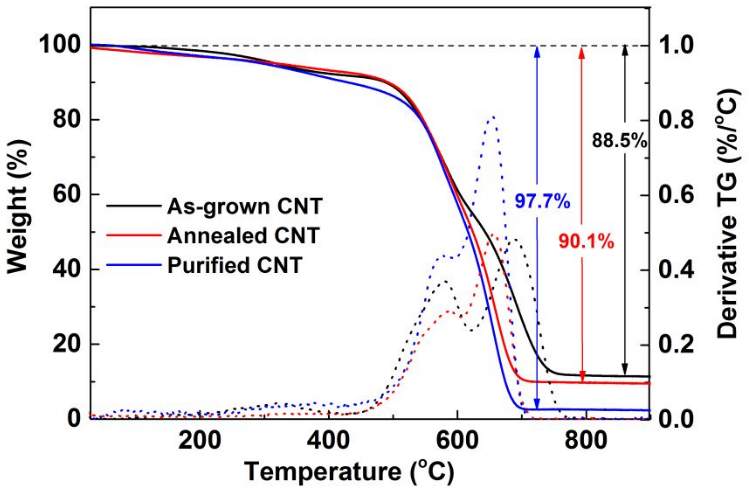

Figure S2. The TGA spectra. TGA spectra of the as-grown, annealed and purified CNT. The dash lines instead of the derivative TG of CNT with temperature.

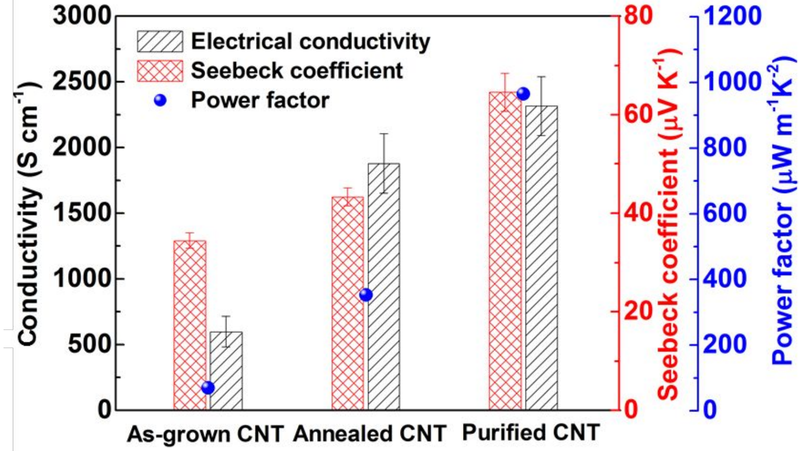

Figure S3. Thermoelectric properties of CNT veils. The electrical conductivities, Seebeck coefficients and power factor of the as-grown, annealed, and purified (acid-washed after annealed) CNT veils at room temperature.

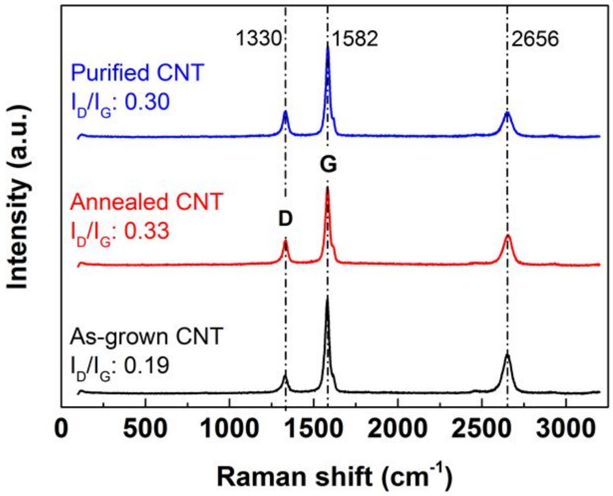

Figure S4. The normalized Raman spectra. The normalized Raman spectra excited with a 633 nm laser for the as-grown, annealed, and purified CNT films. The Raman spectrum of CNT film shows characteristic peaks of D band and G band at ~1,330 cm^{-1} and ~1,582 cm^{-1}, respectively. Because the D-band refers to the disordered crystalline structure or defects in carbon materials, and the G-band contributes to the regular sp2 carbon atoms in the graphitic area, [1-2] the intensity ratios of D and G band (I_D/I_G) refers to the disordered carbon content in the materials. The I_D/I_G ratios increased slightly from 0.19 to 0.33 after the first annealing, and to 0.30 of the followig acid treated CNTs (Purified CNTs).

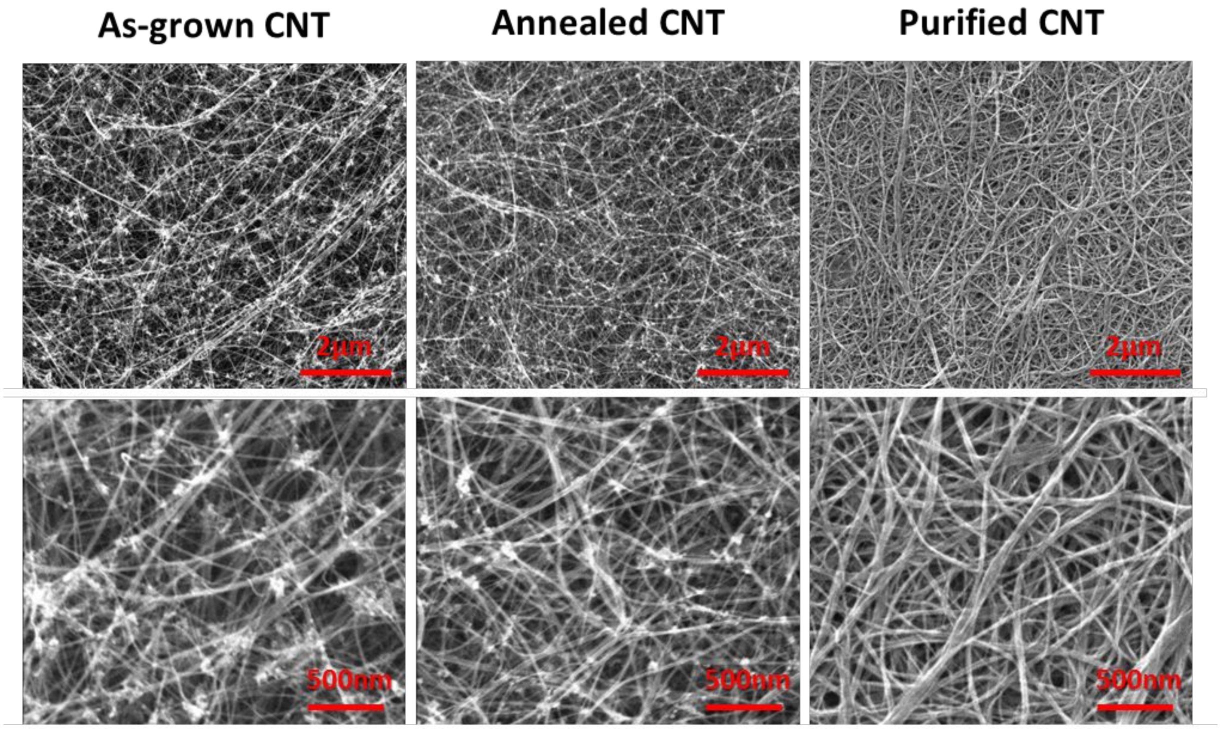

Figure S5. SEM images of as-grown, annealed and purified CNT veils.

Section 2 Optimization of the p and n type doping process

The p-type and n-type doping of CNT veils have been achieved by doping $FeCl_3$ and PEI, respectively, with ethanol as solvent to promote the dopant infiltration into these hydrophobic CNT networks. The charge transfer from CNT valence band to the $FeCl_3$, increases its hole concertration hence lead to a higher positive seebeck value (p-type). On the other hand, Amine-rich PEI molecules act as highly effective electron donors, leading to electrons transfer from PEI to CNTs which increases its electron carriers and turns CNTs into n-type.

As mentioned earlier, it was found that the highly condensed nature of these purified CNT veils has significantly hindered the infiltration process of dopants. For example, it resulted in the residual positive Seebeck value (~20 μ K^{-1}) even after the veils had been immersed in the PEI ethonal solution overnight.

Therefore, only the annealed CNT veils have been used for the subsequent experiments, with a systematic study performed (**Figure S6**) to determe the optimum n- and p-type doping levels. 10 mM $FeCl_3$ doped p-type CNT veil with enhanced electrical conductivity (to 1897 S cm^{-1}), seebeck coefficient (to 60 μV K^{-1}) and thus a power factor of 688 μW $m^{-1}K^{-2}$ has been used after the optmisation process. The optimised n-type CNT veil was obtained at the PEI concentration of 20 mM, with electrical conductivity 1532 S cm^{-1} and Seebeck value of -70 μV K^{-1} . The power factor

of n-type CNT veil reaches 741 μW $m^{-1}K^{-2}$ at room temperature, which is close to the optimized results of n-type CNT reported in literatures (ref) [3-6].

It is worth noting that the p-type doing process has also increased the air stability of the CNTs, with the Seebeck coefficient increased less than 2 μV K^{-1} after exposed in the air environment for 7 months, compared to that of 13 μV K^{-1} from the neat sample (**Figure 1b**). This is attributed the saturation of $FeCl_3$ doping hence limited number of free electrons available within the CNT veils, eliminating the possibility of oxygen doping from the environments to introduce more holes. Good stability was also achieved with n-type doped CNT veils with only 1 μV K^{-1} decrease after 7 months, thanks to the formation of uniform PEI coating layer around CNTs. Additionally, PEI adsorbed on the surface of the CNT has not disrupt the intrinsic CNT structure, but only thicken CNT bundles with PEI concentration increasing (**Figure S7**).

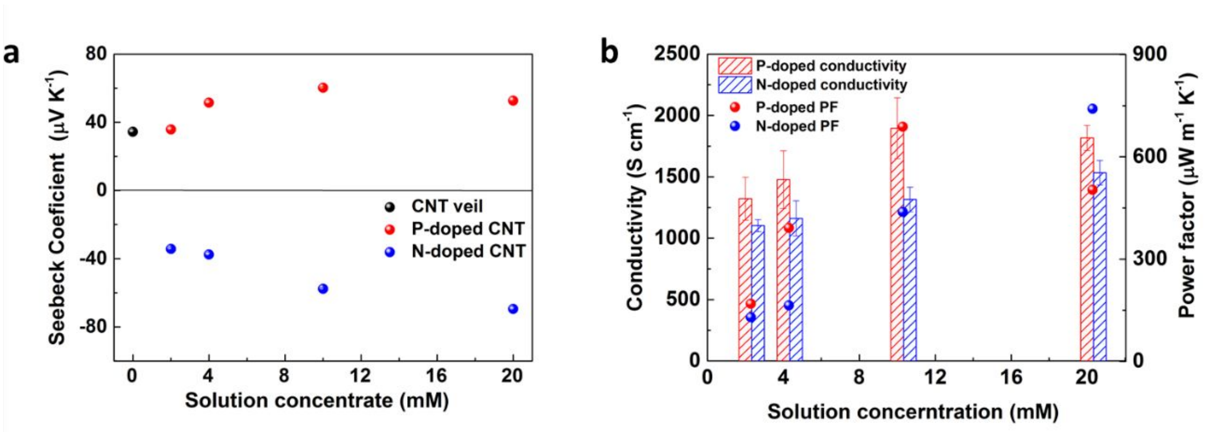

Figure S6. Thermoelectric properties of doped CNT veils. (a) The electrical conductivities, (b) Seebeck coefficients and power factor of the anealled and doped CNT veils at room temperature. The dopant is a solution of PEI and $FeCl_3$ in ethanol with varying concentrations ranging from 2 mM to 20 mM. At low dopant concerntration (2mM), doped oxygen removal is prior to the seebeck coefficient reduction. With the concertraion increasing, $FeCl_3$ dopant effect surpassed it and enhaced both electrical conductivity (to 1897 S cm^{-1}) and seebeck coefficient (to 60 μV K^{-1}) till 10 mM, but saturated at higher concertration (20mM). With the PEI concentration increasing, both the electrical conductivity and negative Seebeck coefficient increase, resulting from the more electron injection.

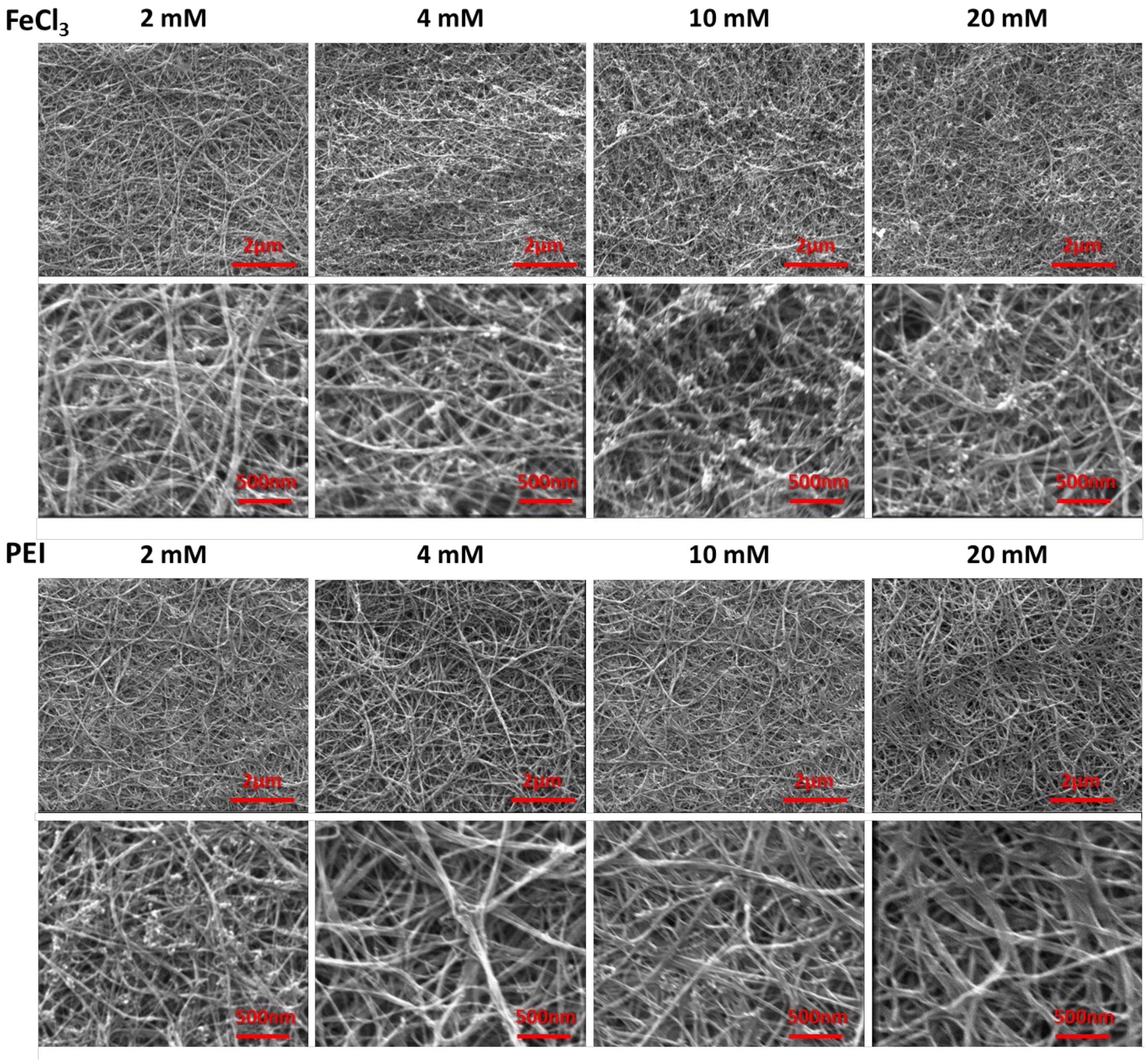

Figure S7. SEM images of doped CNT veils. CNT veils doped by $FeCl_3$ and PEI in ethanol solutions with varying concentrations ranging from 2 mM to 20 mM.

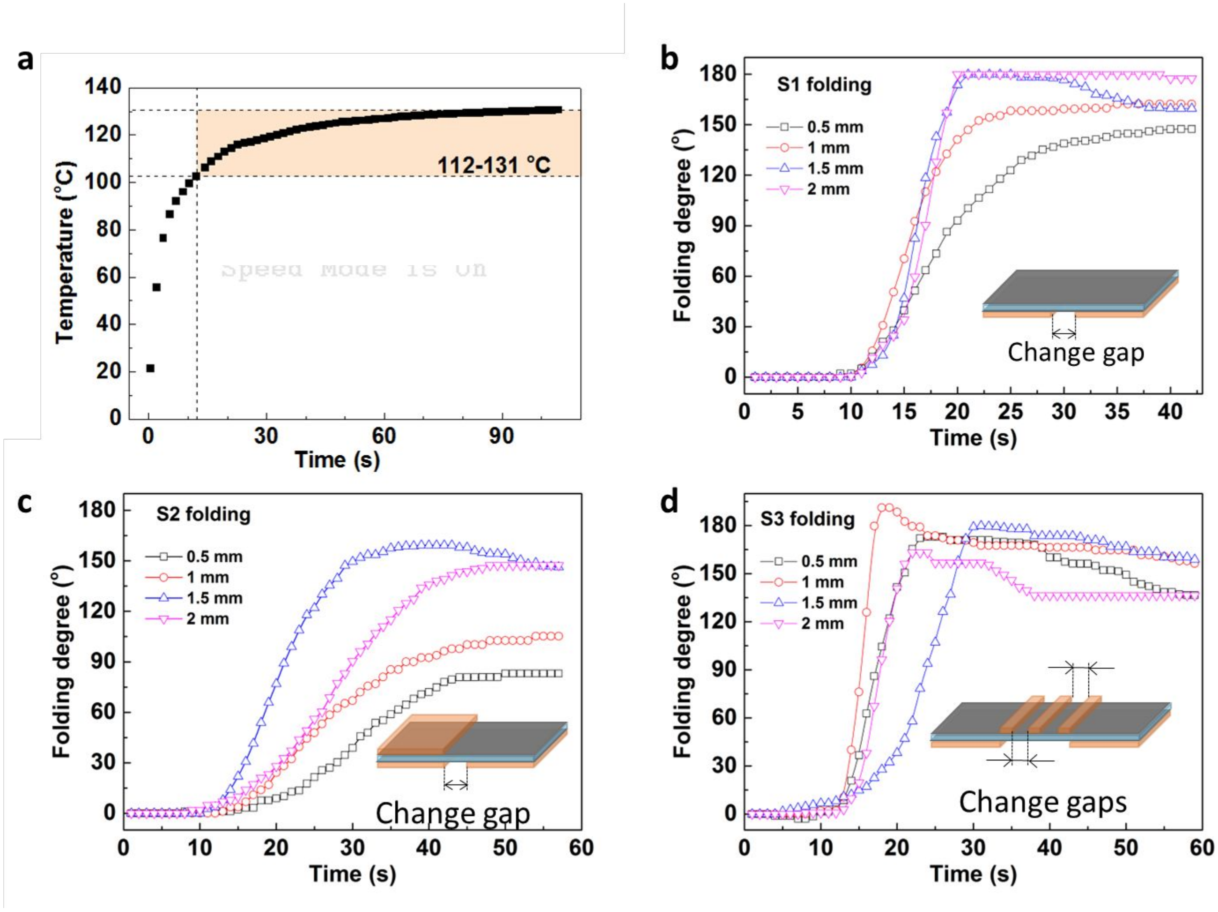

Figure S8. The self-folding angle with various gap widths. (a) The samples' temperature change with the function of time after placed in the oven at 130°C, recorded by IR camera with the folding start time and temperature marked by dash lines. The folding angles change with time and gaps' width for three designs of (b) S1, (c) S2 and (d) S3.

Note: When the sample is kept longer in the oven (more than 30s), the 're-open up' (the decrease of the folding angle) could happen (**Figure S8b** 1.5 mm gap width sample). This phenomenon could be attributed to the two-opposite sides of the fold that have not shrunk at the beginning of

the heating because they have been constrained on one side. When kept more than about 30s, both opposite side of the constrained active layer shrink gradually through the thickness, resulting in an opposite curvature compared to the hinge folding.

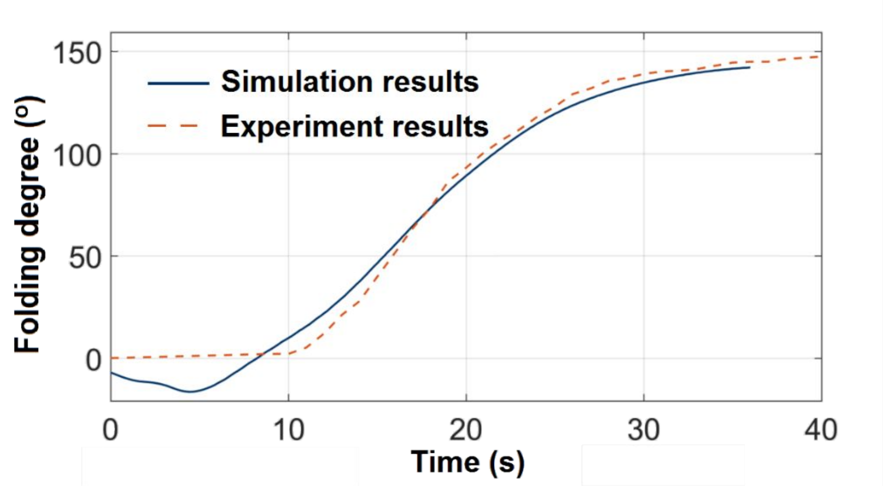

Figure S9 Comparasion between simulation and experimental results on the temperature induced self-folding process. It is interesting to note that simulation results initially report a negative rotation consequence of the thermal expansion of the PS between 0°C and 100°C. This did not take place during the experiment since the structure was placed on a rigid substrate and this predicted negative rotation did not have enough actuation force to lift the structure.

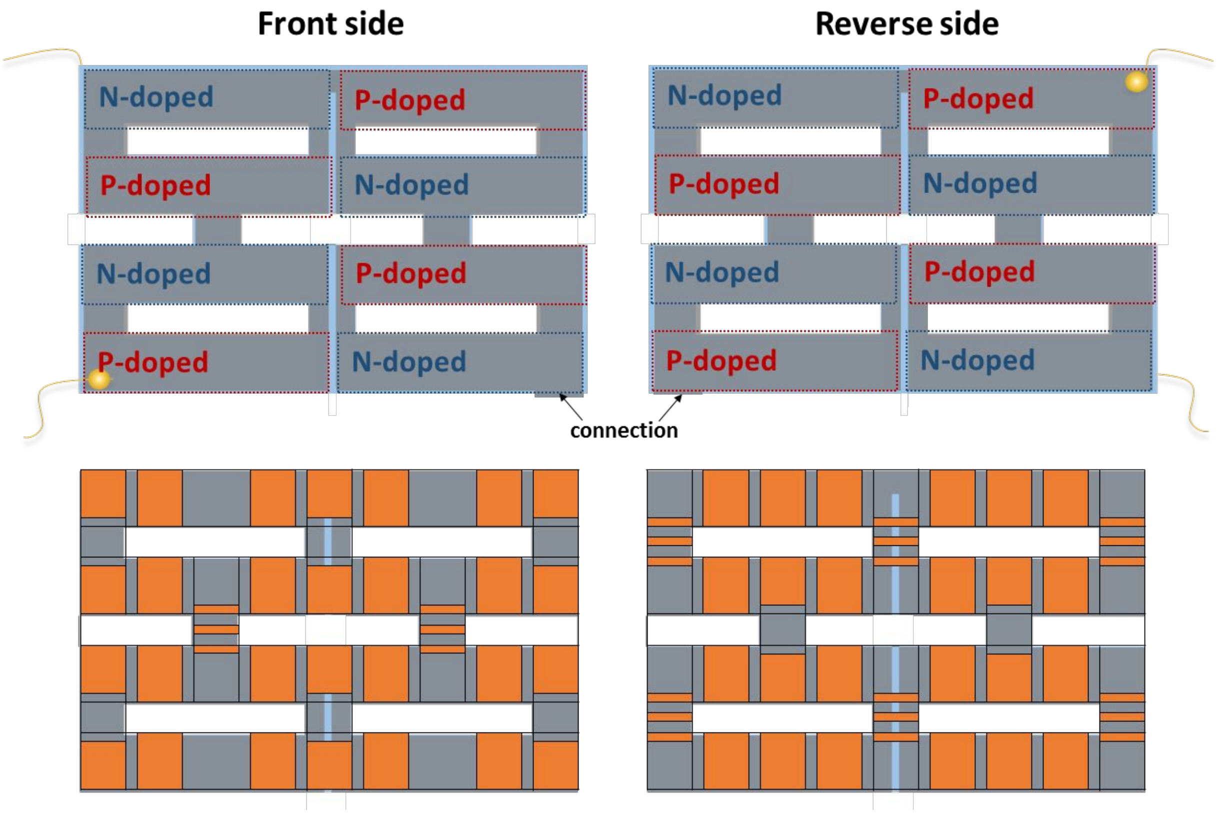

Figure S10. Schematic illustrations of the design for 4-cell honeycomb structure TE module. The p- and n- doped CNT veils (grey) are connected and attached on 2 sides of b-PS, followed by the patterns of PC patches (orange) adhesion. After self-folding process, a 4-cell honeycomb structure TE module, with 8 couples of p-n legs can be formed in the structure connected by 2 electrodes at the corner for measurements (yellow).

Section 3 Normalize power output calculataion

The power output of the self-folded device of the 4-cell (8 thermocouple)device were calculated as following. As shown in Figure 3a, with the P&n type alternatively doped CNT veils covered the both sides of the b-PS layers. Therefore, based on the design pattern (figure S10 and S11), the TE materials area can be calculated as

$$\text{The CNT veil area} = 2 \times (S1 - S2 \times 5 - S3 \times 2)$$

Where the S1, S1, S3 are marked as the following figure. And in this presented case, the CNT veils used in the 4 cell is ($2 \times (80 \times 59.5 - (28 \times 6.5) \times 5 - (36 \times 6.5)) =$) 7232mm^2

Based on the CNT veil's per area density (measured as 5.5g m^{-2}), the weight of the CNT veil used for 4 cell device is 0.4 mg.

According to the power output of 4-cell device power output is ~800nW at ΔT~ 17°C, therefore, the power density calculated as 6.94 mW g^{-1} K^{-2}.

To normalize their power generation performance by the per unit occupied area, the whole 4-cell device could be simplified as a rectangular with length of 35.7mm and width of 27.7mm from a top-side view. Therefore， the power density calculated as 2.82 μW m^{-2} K^{-2}.

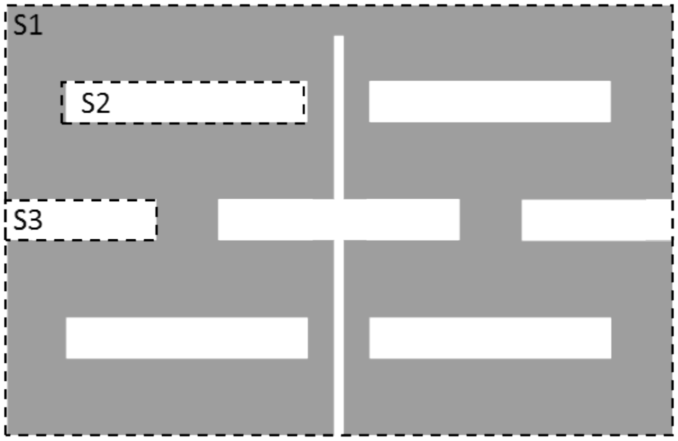

Figure S11. Schematic illustrations of the CNT veils aera for 4-cell honeycomb structure on one side.

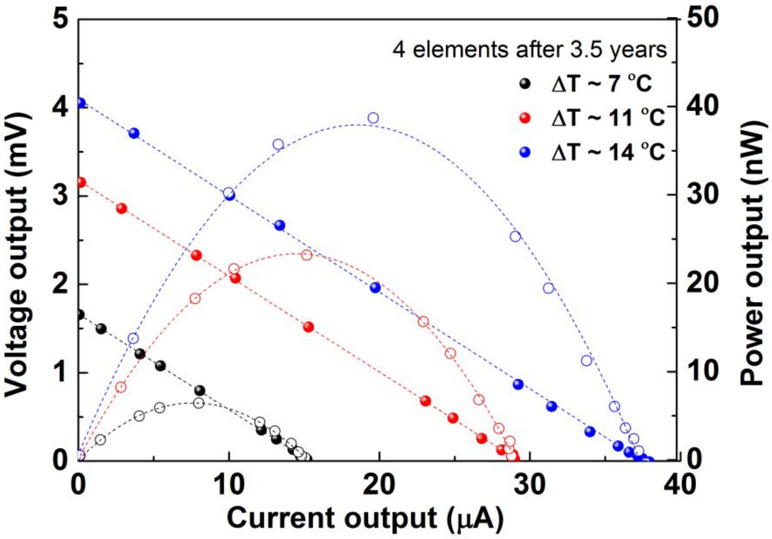

Figure S12. TE performance of the CNT honeycomb structures after 3.5 years. The voltage and power output from 4-themocouples honeycomb TE module consists of a single thermal couple at various temperature differences, with peak output of 4.2 mV and 40 nW at ΔT ~ 14 º.

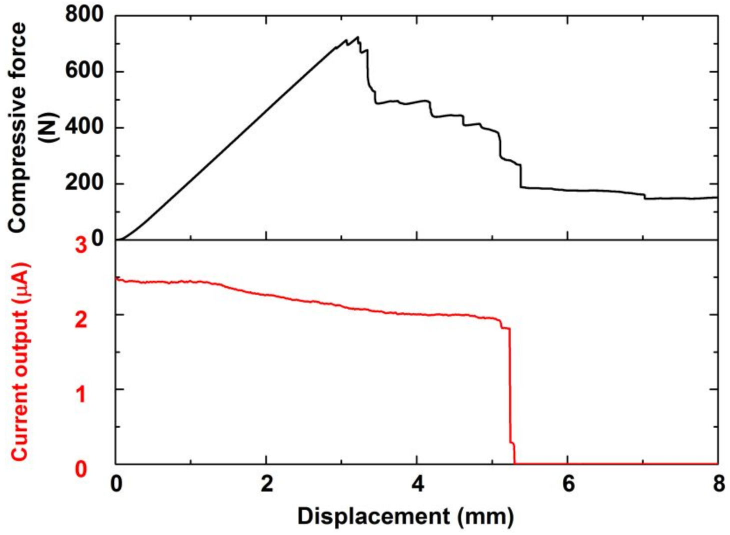

Figure S13. Repeated self-powered sensing results based on the same set up via thermal energy harvesting (17 °C).

REFERENCES

1. Xu, D.; Xu, Q.; Wang, K.; Chen, J.; Chen, Z., Fabrication of Free-standing Hierarchical Carbon Nanofiber/Graphene Oxide/Polyaniline Films for Supercapacitors. *ACS Appl Mater Interfaces* 2014, *6* (1), 200-209.

2. Tai, Z.; Yan, X.; Lang, J.; Xue, Q., Enhancement of Capacitance Performance of Flexible Carbon Nanofiber Paper by adding Graphene Nanosheets. *Journal of Power Sources* 2012, *199*, 373-378.

3. Cho, C.; Culebras, M.; Wallace, K. L.; Song, Y.; Holder, K.; Hsu, J.-H.; Yu, C.; Grunlan, J. C., Stable n-type Thermoelectric Multilayer Thin Films with High Power Factor from Carbonaceous Nanofillers. *Nano Energy* 2016, *28*, 426-432.

4. Wu, G.; Zhang, Z.-G.; Li, Y.; Gao, C.; Wang, X.; Chen, G., Exploring High-Performance n-Type Thermoelectric Composites Using Amino-Substituted Rylene Dimides and Carbon Nanotubes. *ACS Nano* 2017, *11* (6), 5746-5752.

5. Choi, J.; Jung, Y.; Yang, S. J.; Oh, J. Y.; Oh, J.; Jo, K.; Son, J. G.; Moon, S. E.; Park, C. R.; Kim, H., Flexible and Robust Thermoelectric Generators Based on All-Carbon Nanotube Yarn without Metal Electrodes. *ACS Nano* 2017, *11* (8), 7608-7614.

6. Zhou, W.; Fan, Q.; Zhang, Q.; Cai, L.; Li, K.; Gu, X.; Yang, F.; Zhang, N.; Wang, Y.; Liu, H.; Zhou, W.; Xie, S., High-performance and Compact-designed Flexible Thermoelectric Modules enabled by a Reticulate Carbon Nanotube Architecture. *Nat. Commun.* 2017, *8* (1), 14886.
